# Colchicine Impacts Leukocyte Trafficking in Atherosclerosis and Reduces Vascular Inflammation

**DOI:** 10.3389/fimmu.2022.898690

**Published:** 2022-07-04

**Authors:** Ulrike Meyer-Lindemann, Carina Mauersberger, Anna-Christina Schmidt, Aldo Moggio, Julia Hinterdobler, Xinghai Li, David Khangholi, Jan Hettwer, Christian Gräßer, Alexander Dutsch, Heribert Schunkert, Thorsten Kessler, Hendrik B. Sager

**Affiliations:** ^1^ Department of Cardiology, German Heart Centre Munich, Technical University Munich, Munich, Germany; ^2^ DZHK (German Centre for Cardiovascular Research), Partner Site Munich Heart Alliance, Munich, Germany

**Keywords:** atherosclerosis, innate immunity, vascular inflammation, anti-inflammatory treatment, colchicine, leukocyte recruitment, monocytes/macrophages, neutrophils

## Abstract

**Background:**

Inflammation strongly contributes to atherosclerosis initiation and progression. Consequently, recent clinical trials pharmacologically targeted vascular inflammation to decrease the incidence of atherosclerosis-related complications. Colchicine, a microtubule inhibitor with anti-inflammatory properties, reduced cardiovascular events in patients with recent acute coronary syndrome and chronic coronary disease. However, the biological basis of these observations remains elusive. We sought to explore the mechanism by which colchicine beneficially alters the course of atherosclerosis.

**Methods and Results:**

In mice with early atherosclerosis (*Apoe^-/-^* mice on a high cholesterol diet for 8 weeks), we found that colchicine treatment (0.25 mg/kg bodyweight once daily over four weeks) reduced numbers of neutrophils, inflammatory monocytes and macrophages inside atherosclerotic aortas using flow cytometry and immunohistochemistry. Consequently, colchicine treatment resulted in a less inflammatory plaque composition and reduced plaque size. We next investigated how colchicine prevented plaque leukocyte expansion and found that colchicine treatment mitigated recruitment of blood neutrophils and inflammatory monocytes to plaques as revealed by adoptive transfer experiments. Causally, we found that colchicine reduced levels of both leukocyte adhesion molecules and receptors for leukocyte chemoattractants on blood neutrophils and monocytes. Further experiments showed that colchicine treatment reduced vascular inflammation also in post-myocardial infarction accelerated atherosclerosis through similar mechanisms as documented in early atherosclerosis. When we examined whether colchicine also decreased numbers of macrophages inside atherosclerotic plaques by impacting monocyte/macrophage transitioning or *in-situ* proliferation of macrophages, we report that colchicine treatment did not influence macrophage precursor differentiation or macrophage proliferation using cell culture experiments with bone marrow derived macrophages.

**Conclusions:**

Our data reveal that colchicine prevents expansion of plaque inflammatory leukocytes through lowering recruitment of blood myeloid cells to plaques. These data provide novel mechanistic clues on the beneficial effects of colchicine in the treatment of atherosclerosis and may inform future anti-inflammatory interventions in patients at risk.

## Introduction

A large body of evidence has demonstrated that inflammation contributes to all stages of atherosclerotic plaque formation (1–3). Atheroma initiation is characterized by hyperlipidemia-activated endothelial cells, which upregulate leukocyte adhesion molecules and leukocyte attracting chemokines. Consequently, leukocytes – the effector cells of the immune system – are recruited from the blood stream and accumulate in the arterial intima. Intimal leukocytes, mainly myeloid cells (neutrophils, monocytes and macrophages), fuel plaque initiation and progression through 1) release of pro-inflammatory cytokines and chemokines, 2) lipid core formation and enlargement, and 3) thinning of the fibrous cap. As a consequence, plaque destabilization with fibrous cap erosion/rupture may occur resulting in complications such as myocardial infarction (MI) or stroke. Given this background, clinical studies recently probed whether therapeutic targeting of inflammatory pathways may be beneficial in prevention and treatment of atherosclerosis (4). Indeed, the Canakinumab Anti-inflammatory Thrombosis Outcomes Study (CANTOS) provided the first proof of the contribution of inflammation to atherosclerosis in humans and demonstrated that neutralizing interleukin-1 beta (IL-1β) reduces cardiovascular events in patients with atherosclerosis and prior MI (5, 6). This concept was further reinforced by two recent trials using the anti-inflammatory drug colchicine (7, 8): In the Colchicine Cardiovascular Outcomes Trial (COLCOT), colchicine led to a significantly lower risk of ischemic cardiovascular events in patients with prior MI (7) while in the Low-Dose Colchicine (LoDoCo) 2 trial, it reduced cardiovascular events in chronic coronary disease patients (8). Colchicine, an orally administered plant alkaloid used to treat gout and pericarditis, is a potent anti-inflammatory agent mainly acting by inhibiting tubulin assembly and suppressing microtubule formation (key components of the cellular cytoskeleton), but also interfering with NLRP3 (NACHT, LRR and PYD domains-containing protein 3)-inflammasome formation and thereby reducing the release of activated pro-inflammatory IL-1β and interleukin-18 (IL-18) (9).

Although large randomized trials using colchicine to treat atherosclerosis showed promising results, we do not completely understand the biological basis of the beneficial effects. In this study, we sought to explore how colchicine prevents progression of atherosclerosis. Our results show that colchicine reduces vascular inflammation by dampening uptake of inflammatory leukocytes into plaques through altering the recruitment profile of circulating monocytes and neutrophils.

## Materials and Methods

### Mouse Studies

*Apoe^-/-^* (B6.129P2-Apoetm1Unc/J) and *Ubc-GFP* mice (C57BL/6-Tg(UBC-GFP)30Scha/J) were purchased from the The Jackson Laboratory (Bar Harbor, ME, USA) and expanded by in-house breeding. C57BL/6 J mice were purchased from Charles River Laboratories (Sulzfeld, Germany). For experiments, *Apoe^-/-^* mice were fed a high cholesterol diet (HCD, 21.2% fat by weight and 0.2% cholesterol, TD.88137, Envigo, Indianapolis, IN, USA) for a total period of eight weeks. During the last four weeks of HCD, a sterile solution of vehicle [phosphate buffered saline (PBS)] or colchicine (dissolved in sterile PBS, 0.25 mg/kg bodyweight (BW); C3915, Sigma Aldrich, St. Louis, MO, USA) was injected intraperitoneally once daily. The colchicine dosage used was based on previously published studies (10, 11). Unfortunately, it was not possible to quantify plasma concentrations of colchicine in our mice.

For MI experiments, *Apoe^-/-^* mice were fed a HCD for four weeks and anaesthetized with a combination of midazolam (5.0 mg/kg BW), medetomidine (0.5 mg/kg BW) and fentanyl (0.05 mg/kg BW). C57BL/6 J mice were anaesthetized without prior HCD treatment. After thoracotomy in the left intercostal space, the left anterior descending coronary artery was located and permanently occluded using an 8-0 prolene suture to induce MI. For analgesia, mice received buprenorphine every 8 hours for 3 days. *Apoe^-/-^* mice were fed the HCD for an additional 5 weeks, with PBS or colchicine injections for the last 4 weeks as described above, while C57BL/6 J mice received vehicle or colchicine (0.25 mg/kg BW every 8 hours) during the first three days after MI.

For all experiments, age- and sex-matched littermates at 8 to 12 weeks of age were used. Assignment to groups was random. Animal experiments were conducted in accordance with the German legislation on protection of animals and approved by the local animal care committee (AZ: ROB-55.2-2532.Vet_02-16-92).

### Tissue Processing

For *in vivo* staining of circulating blood leukocytes, an antibody directed against CD45-BV605 (clone 30-F11, 1:10 in 100 µl PBS, BioLegend, San Diego, CA, USA) was injected intravenously 5 min before euthanizing the animals under isoflurane anesthesia. Blood samples were obtained by retrobulbar collection into EDTA-coated tubes. Lysis of erythrocytes was performed in blood samples in 1X RBC Lysis Buffer (420302, BioLegend). After stopping the reaction with PBS, samples were centrifuged at 400 g for 10 min at 4°C and resuspended in FACS Buffer (PBS containing 0.5% bovine serum albumin, A2153, Sigma Aldrich). Bone marrow was sampled from femurs by flushing the bones with PBS. Cells were then filtered using a 40 µm cell strainer.

For analysis of aortic plaques and cardiac leukocytes, hearts were perfused through the left ventricle with PBS, aortas were extracted carefully from root to common iliac artery bifurcation removing the surrounding tissue. Hearts and aortas were minced in respective digestion buffer. Then, tissues were digested in a solution composed of collagenase I (450 U/ml, C0130), collagenase XI (125 U/ml, C7657), DNase I (60 U/ml, D5319-500UG), and hyaluronidase (60 U/ml, H3506, all Sigma Aldrich) in PBS for 1 h under agitation at 37°C.

For flow cytometric analysis of aortic endothelial cells, digestion buffer consisted of 1X PBS containing DNase I (250 U/ml) and collagenase IV (2500 U/ml, LS004212, CellSystems, Troisdorf, Germany) and aortas were digested for 40 min under agitation at 37°C.

Subsequently, cell suspensions were filtered using a 40 µm cell strainer and resuspended in FACS buffer.

### Flow Cytometry and Fluorescence Activated Cell Sorting

Aortic and cardiac cell suspensions were stained with antibodies labelling murine hematopoietic lineage markers (B220 (clone RA3-6B2); CD90.2 (clone 53-2.1, 1:300 dilution); CD49b (clone DX5, 1:1200 dilution); NK1.1 (clone PK136); Ter-119 (clone TER-119) and Ly6G (clone 1A8), all conjugated with phycoerythrin (PE)) and antibodies directed against CD45.2 (PerCP/Cy5.5-conjugated, clone 104, 1:300); CD11b (APC/Cy7-conjugated, clone M1/70); F4/80 (PE/Cy7-conjugated, clone BM8); and Ly6C (BV421- or FITC-conjugated, clone HK1.4). For blood and bone marrow analysis, an antibody against CD115 (BV711-conjugated, clone AFS98) was added to the staining mixture. For BrdU experiments, cells were subsequently fixed and permeabilized and additionally stained in an APC-conjugated anti-BrdU antibody according to the manufacturer’s recommendations (552598, BD Biosciences). All antibodies were used in 1:600 dilutions and purchased from BioLegend unless indicated otherwise.

Aortic endothelial cell samples were stained with antibodies against CD54-APC (ICAM1) (clone YN1/1.7.4, 1:600 dilution); CD102-biotin (ICAM2) (clone 3C4, 1:600 dilution; streptavidin-BV510 (1:600 dilution) was used for secondary labelling)); CD106-PE/Cy7 (VCAM1) (clone 429, 1:100 dilution); CD62E-PE (E-Selectin) (clone 10E9.6, 1:50 dilution, BD Biosciences, San Jose, CA, USA); CD62P-FITC (P-Selectin) (clone RB40.34, 1:300 dilution, BD Biosciences); CD31-BV421 (clone 390, 1:600 dilution); CD107a-APC/Cy7 (LAMP1) (clone 1D4B, 1:300 dilution); and CD45.2-PerCP/Cy5.5 (clone 104, 1:300 dilution, all BioLegend unless indicated otherwise).

Cell suspensions were submitted to flow cytometric analysis on a BD LSRFortessa (BD Biosciences) and files were analyzed using FlowJo software (version 9 or 10). Therefore, cells were pre-gated on viable (FSC-A vs. SSC-A) and single (FSC-A vs. FSC-W and SSC-A vs. SSC-W) cells. Myeloid cells were characterized as follows: neutrophils as CD45.2^high^CD11b^high^lineage^high^CD115^low^Ly6C^intermediate^, monocytes as CD45.2^high^CD11b^high^lineage^low^F4/80^low^Ly6C^high/low^ (in aortic cell suspensions) or CD45.2^high^CD11b^high^lineage^low^CD115^high^Ly6C^high/low^ (in blood and bone marrow) and macrophages as CD45.2^high^CD11b^high^lineage^low^Ly6C^low/intermediate^F4/80^high^. Contaminating blood leukocytes were excluded by omitting CD45-BV605^+^ cells in the gating. Endothelial cells were identified as CD45.2^low^CD31^high^CD107a^intermediate/high^ and adhesion molecule expression was quantified using respective histograms.

For fluorescence-activated cell sorting (FACS), blood leukocytes and aortic endothelial cells were sorted on a FACS Aria III using FACSDiva Software version 6 (BD Biosciences). Neutrophils were identified as CD45.2^high^CD11b^high^lineage^high^CD115^low^Ly6C^intermediate^, Ly6C^high^ and Ly6C^low^ monocytes as CD45.2^high^CD11b^high^lineage^low^CD115^high^Ly6C^high/low^, and endothelial cells as CD45.2^low^CD31^high^ cells. Cells were directly sorted into RNA extraction buffer (KIT0204, Thermo Fisher Scientific) containing tubes and snap-frozen to -80°C.

### Histology

Aortic roots were excised, embedded in optimal cutting temperature compound (Sakura Finetek, Tokyo, Japan) and snap frozen to -80°C. Samples were cut into 5 µm cross-sections. Per staining, 5 sections were examined at 25 µm intervals per mouse.

The absolute size of the plaques was determined by Masson trichrome staining (HT15-1KT, Sigma Aldrich). Sections were fixed in 4% PFA for 45 seconds and stained corresponding to the manufacturer’s protocol. Mean total plaque area [in µm (2)] was evaluated for sections showing at least two complete cusps and analyzed using ImageJ. An average value per mouse was then calculated.

To analyze the distribution of CD11b^+^ cells, sections were fixated in ice-cold acetone for 10 min and blocked in 10% rabbit serum (Vector Laboratories, Burlingame, CA, USA). Specimens were stained with an anti-CD11b antibody (101202, BioLegend) or anti-Ly6G antibody (clone 1A8, BioLegend), respectively, followed by a HRP conjugated secondary antibody (ab6734) and AEC substrate (ab64252, both abcam, Cambridge, MA, USA). Cell nuclei were counterstained with Gill’s hematoxylin solution II (1051752500, Merck Millipore). The CD11b or Ly6G content was determined by quantifying the CD11b- or Ly6G-positive area per total plaque area using ImageJ software.

### Isolation of Nucleic Acids and Real-Time Quantitative Polymerase Chain Reaction (qPCR)

Aortic arches were minced in 500 µl of Qiazol lysis reagent (Qiagen, Hilden, Germany) with the aid of a mechanical disruptor (TH220) using soft tissue tips (all OMNI International, Kennesaw, GA, USA). Total ribonucleic acid (RNA) was extracted by using the RNeasy Mini Kit (Qiagen) corresponding to the manufacturer´s instructions, involving an additional DNA removal step (RNase-free DNase set, Qiagen).

RNA of FACS-sorted leukocytes and aortic endothelial cells was isolated according to the manufacturer’s instructions (PicoPure™ RNA Isolation Kit, Thermo Fisher Scientific).

RNA quality was assessed using a NanoQuant Plate on an Infinite M200 PRO plate reader (both TECAN, Männedorf, Switzerland) and first-strand cDNA was generated using the High-Capacity RNA-to-cDNA kit (Applied Biosystems, Waltham, MA).

Real-time qPCR was performed using TaqMan probes (listed in **Supplementary Table S1**) and TaqMan Fast Universal PCR Master Mix (4352042) over 40 cycles on a ViiA 7 system (all Thermo Fisher Scientific, Waltham, MA, USA). *Gapdh* was used as a housekeeping gene and data were converted to 2^-△△Ct^ values.

### Cell Sorting and Adoptive Transfer

*Ubc-GFP* donor animals were subjected to the experiments either untreated or after one week of treatment with PBS or colchicine (0.25 mg/kg BW) injected intraperitoneally once daily. Single cell suspensions were prepared from the bone marrow as described above. Neutrophils and monocytes were isolated by staining the cells with Ly6G-PE (clone 1A8) and CD115-biotin (clone AFS98, both BioLegend) followed by linking to magnetic beads (anti-PE and streptavidin microbeads, Miltenyi Biotec, Bergisch Gladbach, Germany) and enrichment on magnetic-activated specific columns (130-042-401, Miltenyi Biotec). The degree of purification of the separated cells was determined by flow cytometry.

Equal cell amounts were injected intravenously into *Apoe^-/-^* mice 24 h before harvest. Quantification of recruited CD11b^high^GFP^high^ cells within aortas was assessed by flow cytometry.

### Cell Culture

Bone marrow cells were isolated from C57BL/6 J mice by flushing the femurs with RPMI 1640 (A1049101, Thermo Fisher Scientific) containing 2% of fetal bovine serum (FBS; S0615, Sigma Aldrich). Erythrocytes were removed by incubating the cells in 1X RBC lysis buffer (BioLegend). Subsequently, cells were cultured for the indicated time in RPMI 1640 containing 10% FBS, 100 U/ml penicillin-streptomycin (15140122, Thermo Fisher Scientific) and 50 ng/ml recombinant mouse M-CSF (R&D Systems, Minneapolis, MN, USA) in 6-well plates [80,000 cells/cm (2)] in a humidified incubator with 5% CO_2_ at 37°C. Fresh medium was added every day, and every third day the medium was partially replaced with fresh medium containing the resuspended non-adherent cells. In the respective experiments, colchicine (0, 1, or 10 ng/ml final concentration) was added along with the culture medium daily for one to six days as indicated in the respective figures.

For BrdU experiments, bone marrow cells were cultured into macrophages in 100 mm dishes for two or three days, respectively, and reseeded in 6-well plates [80,000 cells/cm (2)]. Colchicine (0, 1, or 10 ng/ml final concentration) was added along with fresh culture medium for the last 24 or 48 hours as indicated in the respective figures. BrdU solution (10 µM final concentration, BrdU Flow Kit, BD Biosciences) was added two hours before harvesting of cells.

Non-adherent cells were collected, adherent cells were washed in HEPES-buffered saline solution (Promocell, Heidelberg, Germany), detached by incubating in Accutase (Sigma Aldrich) and, together with non-adherent cells, resuspended in FACS buffer before flow cytometry analysis.

### Statistical Analysis

All statistical analysis was conducted using GraphPad Prism version 9. Normality distribution was tested by the D’Agostino-Pearson omnibus K2 normality test or the Shapiro-Wilk test for sample sizes n<8. Two-group comparisons were performed using two-sided Student´s *t*-test (normally distributed data; Welch’s *t*-test was used if variances between both groups were significantly different, as tested by *F*-test) or two-sided Mann-Whitney *U*-test (non-normally distributed data), as shown in the figure legends in combination with sample sizes. Comparisons of three or more groups were performed using repeated measures one-way ANOVA followed by Dunnett’s multiple comparisons test (normally distributed data) and Friedman test followed by Dunn’s multiple comparisons test (non-normally distributed data), as appropriate and indicated in the figure legends. A two-sided ROUT´s test was applied to determine statistical outliers. All graphs illustrate data as mean + s.e.m. Statistical significance was assumed if *P*-values were <0.05. Mouse experiments were performed at least twice or with n ≥ 10. Where appropriate, variation between experiments was adjusted by normalizing absolute values to a representative experiment.

## Results

### Colchicine Treatment Reduced Inflammatory Leukocyte Accumulation in Atherosclerotic Aortas

**Figure 1:**
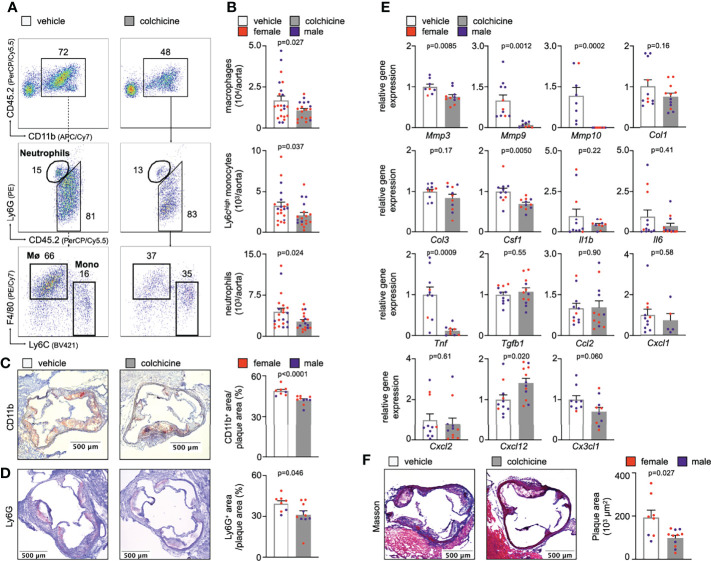
Colchicine treatment reduces plaque inflammation. **(A)** Flow cytometric gating and **(B)** quantification of myeloid cells in atherosclerotic aortas in vehicle- vs. colchicine-treated *Apoe^-/-^* mice (*n*=18-21 per group, 62-67% female, Welch’s *t*-test for macrophages (Mø), Mann-Whitney *U*-test for monocytes (Mono) and neutrophils as appropriate). Numbers next to gates indicate population frequencies (%). Representative immunohistochemical staining for **(C)** myeloid cells (CD11b) and **(D)** neutrophils (Ly6G) of sectioned aortic roots from vehicle- vs. colchicine-treated *Apoe^-/-^* mice (*n*=8-10 per group, 60-75% female, Mann-Whitney *U*-test for CD11b, Student’s *t*-test for Ly6G). Bar graphs show quantification of positive CD11b- and Ly6G-area, respectively. Scale bars represent 500 µm. **(E)** Quantitative real-time PCR for gene expression quantification of fibrotic, inflammatory and cytokine genes in aortas of vehicle- vs. colchicine-treated *Apoe^-/-^* mice (*n*=5-12 per group, 55-88% female, Student’s/Welch’s *t*-test or Mann-Whitney *U*-test as appropriate). *Mmp3/Mmp9/Mmp10* (matrix metalloproteinase-3/9/10), *Col1/Col3* (collagen-1/3), *Csf1* (colony stimulating factor 1), *Il1β* (interleukin 1 beta), *Il6* (interleukin 6), *Tnf* (tumor necrosis factor), *Tgfb1* (transforming growth factor beta 1), *Ccl2* (C-C Motif Chemokine Ligand 2), *Cxcl1* (C-X-C Motif Chemokine Ligand 1), *Cxcl2* (C-X-C Motif Chemokine Ligand 2), *Cxcl12* (C-X-C Motif Chemokine Ligand 12), *Cx3cl1* (C-X3-C Motif Chemokine Ligand 1). Data are presented as mean+s.e.m. **(F)** Representative Masson Trichrome staining and quantification of total plaque area (*n*=8-11 per group, 64-75% female, Welch’s *t*-test). Scale bars represent 500 µm. Dots within bar plots show the gender of the mice with a color code: purple (male) and red (female).

To explore the mechanisms by which colchicine beneficially alters the course of atherosclerosis, we treated atherosclerosis-prone mice (*Apoe^-/-^* mice on a high cholesterol diet for eight weeks) with either vehicle or colchicine for four weeks. We found that colchicine reduced inflammatory leukocyte numbers over vehicle in atherosclerotic aortas (**Figures 1A, B**, **Supplementary Figure 1A, B**). We corroborated our flow cytometry findings using immunohistochemistry and observed fewer intimal myeloid cells (monocytes/macrophages and neutrophils) and neutrophils in aortic root sections from colchicine treated mice stained for the myeloid marker CD11b and the neutrophil marker Ly6G, respectively (**Figures 1C, D**). Next, we tested whether reduced plaque inflammation changed the overall plaque character. Phenotyping of plaques using quantitative polymerase chain reaction (qPCR) revealed that colchicine treatment reduced expression of mRNAs that encode the pro-inflammatory cytokine TNF (tumor necrosis factor), while it had no effect on transcriptional levels of IL-1β (interleukin-1 beta) and IL-6 (interleukin-6) (**Figure 1E**). Levels of MMP (matrix metalloproteinase)-3, MMP-9, and MMP-10 decreased in colchicine treated mice (**Figure 1E**). MMPs support extracellular matrix degradation, a process that may lead to atherosclerotic plaque destabilization (12). Histology revealed a smaller total plaque size in the colchicine group (**Figure 1F**). Of note, colchicine treatment did not alter body weight or LDL cholesterol levels in mice. Colchicine treated mice did not show any type of discomfort or impairment compared to mice receiving vehicle (**Supplementary Figure 2A**). Taken together, these data indicate that colchicine treatment limited plaque inflammation and hence progression of atherosclerosis.

### Colchicine Treatment Dampened Plaque Leukocyte Recruitment

**Figure 2:**
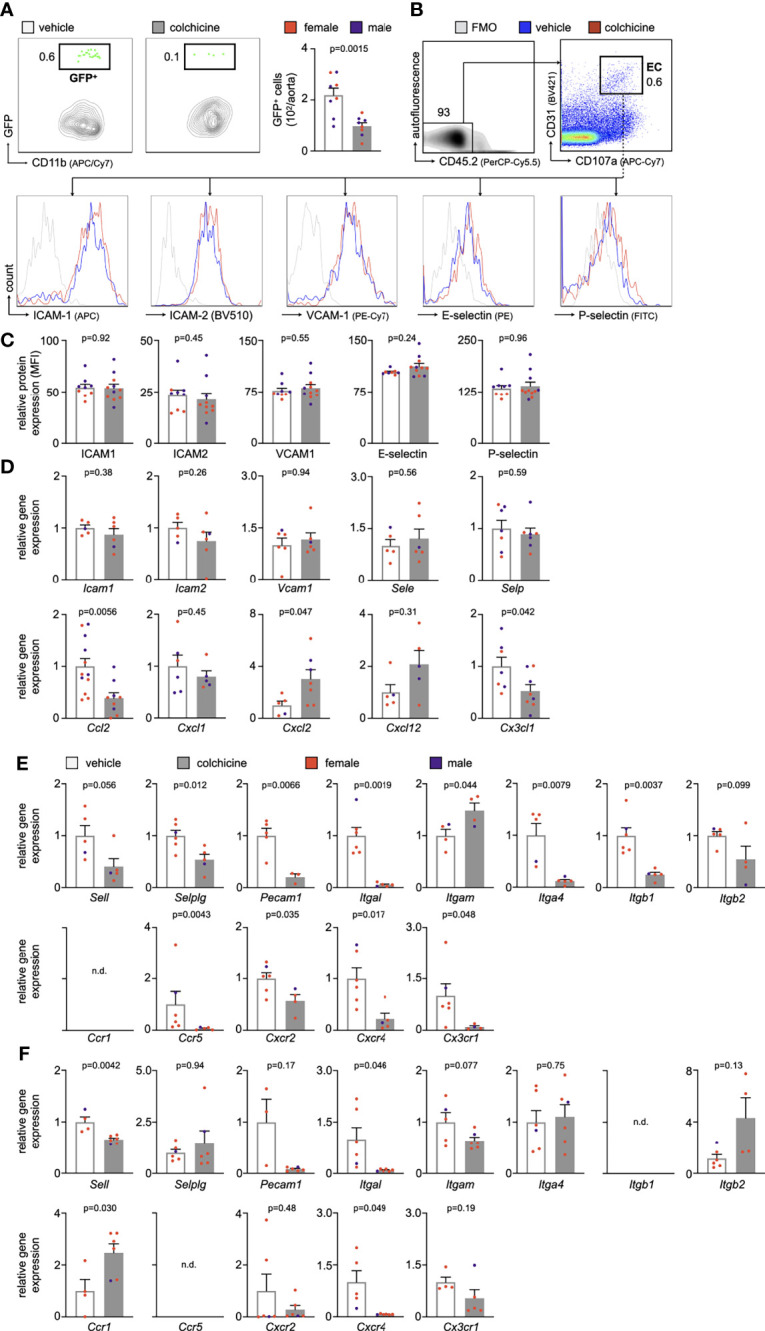
Colchicine treatment dampens leukocyte recruitment to atherosclerotic aortas by silencing neutrophil and monocyte activation. **(A)** Flow cytometric gating and quantification of GFP^high^ (GFP^+^) myeloid cells in atherosclerotic aortas 24h after adoptive transfer of GFP^high^ monocytes and neutrophils into vehicle- vs. colchicine-treated *Apoe^-/-^* mice (*n*=8 per group, 38-50% female, Student’s *t-*test). **(B)** Gating strategy and histograms of leukocyte adhesion molecules on aortic endothelial cells (EC) from vehicle- vs. colchicine-treated *Apoe^-/-^* mice. **(C)** Quantification of mean fluorescent intensities (MFI, representing relative protein levels) of adhesion molecules expressed by aortic endothelial cells from vehicle- vs. colchicine-treated *Apoe^-/-^* mice (*n*=9-11 per group, 44-55% female, Student’s *t*-test or Mann-Whitney *U*-test as appropriate). Bar graphs indicate relative change of MFI standardized to controls. FMO (fluorescence minus one (respective antibody omitted)) control. Quantitative real-time PCR for gene expression quantification in fluorescence-activated cell sorting (FACS)-purified **(D)** aortic endothelial cells, **(E)** blood neutrophils and **(F)** blood Ly6C^high^ monocytes of vehicle- vs. colchicine-treated *Apoe^-/-^* mice (*n*=3-12 per group, 33-100% female, Student’s/Welch’s *t*-test or Mann-Whitney *U*-test as appropriate). ICAM-1/*Icam1* (intercellular adhesion molecule 1), ICAM-2/*Icam2* (intercellular adhesion molecule 2), VCAM-1/*Vcam1* (vascular cell adhesion protein 1), E-selectin/*Sele*, P-selectin/*Selp*, L-selectin/*Sell*, selectin P ligand/*Selplg*, platelet endothelial cell adhesion molecule-1/*Pecam1*, integrin subunit alpha L/*Itgal*, integrin subunit alpha M/*Itgam*, integrin subunit alpha 4/*Itga4*, integrin subunit beta 1/*Itgb1*, integrin subunit beta 2/*Itgb2*, C-C motif chemokine ligand 2/*Ccl2*, C-X-C motif chemokine ligands 1 + 2+12/*Cxcl1/Cxcl2/Cxcl12*, C-X3-C motif ligand 1/*Cx3cl1*, C-C chemokine receptor types 1 + 5/*Ccr1/Ccr5*, C-X-C Motif chemokine receptor types 2 + 4/*Cxcr2/Cxcr4* and C-X3-C Motif Chemokine Receptor 1/*Cx3cr1*. Data are presented as mean+s.e.m. Numbers next to gates indicate population frequencies (%). Dots within bar plots show the gender of the mice with a color code: purple (male) and red (female).

We next addressed how colchicine treatment reduced inflammatory leukocyte accumulation in atherosclerotic aortas. To explore whether colchicine dampened blood inflammatory leukocyte recruitment into atherosclerotic aortas, we performed adoptive transfer experiments. Here, we isolated GFP^high^ myeloid cells (monocytes admixed with neutrophils) from naïve transgenic *Ubc-GFP* mice (all leukocytes express green fluorescent protein, GFP) and injected these cells intravenously into *Apoe^-/-^* mice (all cells are GFP^negative^) which were treated with either vehicle or colchicine for 4 weeks (**Supplementary Figure 1C**). 24h after the transfer, we quantitated GFP^high^ myeloid cells inside atherosclerotic aortas using flow cytometry and found that colchicine treatment lowered GFP^high^ myeloid cell numbers, while GFP^high^ myeloid cell numbers in the blood did not show any differences between the two groups (**Figure 2A**, **Supplementary Figure 1D**). These data show that colchicine mitigates myeloid cell uptake from blood into atherosclerotic aortas.

### Colchicine Treatment Altered the Recruitment Profile of Monocytes and Neutrophils

**Figure 3:**
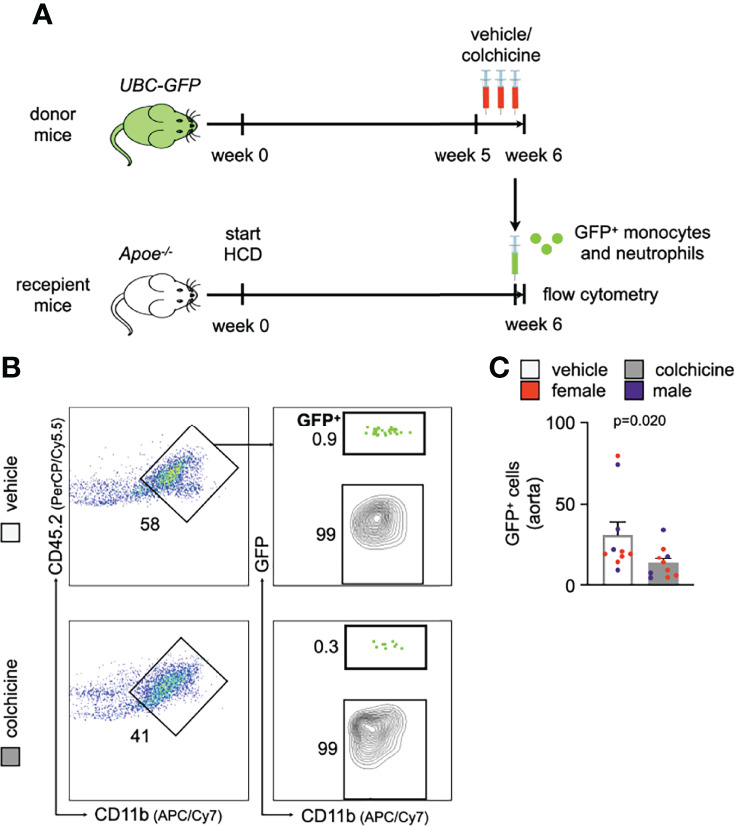
Colchicine exposed neutrophils and monocytes show reduced recruitment capacities. **(A)** Experimental scheme. **(B)** Flow cytometric gating and **(C)** quantification of GFP^high^ myeloid cells (GFP^+^) in atherosclerotic aortas 24h after adoptive transfer of either vehicle- or colchicine-exposed GFP^high^ monocytes and neutrophils into *Apoe^-/-^* mice (*n*=10 per group, 60% female, Mann-Whitney *U*-test). Data are presented as mean+s.e.m. Numbers next to gates indicate population frequencies (%). Dots within bar plots show the gender of the mice with a color code: purple (male) and red (female).

**Figure 4:**
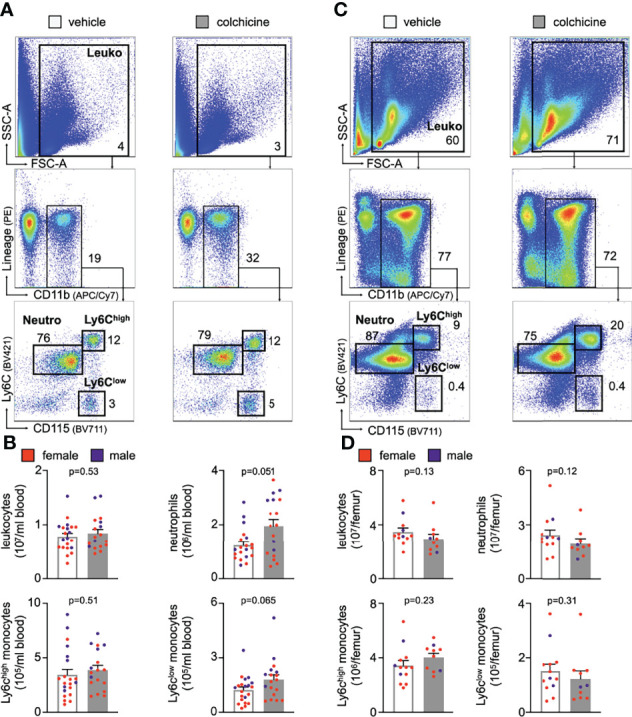
Colchicine treatment did not alter numbers of blood leukocyte subsets. Flow cytometric gating and quantification of myeloid cells in the blood **(A, B)** (*n*=18-21 per group, 62-67% female, Student’s *t*-test for total leukocytes (Leuko) and Ly6C^high^ monocytes (Ly6C^high^), Mann-Whitney *U*-test for neutrophils (Neutro) and Ly6C^low^ monocytes (Ly6C^low^) and bone marrow **(C, D)** in vehicle- vs. colchicine-treated *Apoe^-/-^* mice (*n*=10-13 per group, 77-80% female, Mann-Whitney *U*-test). Data are presented as mean+s.e.m. Numbers inside/next to gates indicate population frequencies (%). Dots within bar plots show the gender of the mice with a color code: purple (male) and red (female).

Leukocyte recruitment refers to a process in which endothelial cells and circulating leukocytes need to interact closely to mediate leukocytes rolling, adhesion and transmigration/extravasation (13, 14). To investigate how colchicine treatment limited myeloid cell recruitment from blood to atherosclerotic aortas, we used flow cytometry to assess levels of leukocyte adhesion molecules on endothelial cells from atherosclerotic aortas. Our data revealed that colchicine had no effect on protein levels of leukocyte adhesion molecules ICAM-1 (intercellular adhesion molecule-1), ICAM-2 (intercellular adhesion molecule 2), VCAM-1 (vascular cell adhesion protein 1), E-selectin and P-selectin (**Figures 2B, C**). Moreover, we found that colchicine treatment lowered transcriptional levels of only Ccl2 (chemokine (C-C motif) ligand 2) and Cx3cl1 (C-X3-C Motif Chemokine Ligand 1) – both known chemoattractants for monocytes – while expression of other leukocyte-attracting chemokines and leukocyte adhesion molecules remained unchanged in fluorescence-activated cell sorting (FACS)-purified endothelial cells from atherosclerotic aortas (**Figure 2D**). Next, we FACS-purified blood Ly6C^high^ monocytes and neutrophils from *Apoe^-/-^* mice treated with either vehicle or colchicine. In contrast to endothelial cells, we found that both blood Ly6C^high^ monocytes and neutrophils underwent profound phenotypic alteration in colchicine treated mice (**Figure 2E, F** **)**. Blood neutrophils responded to colchicine treatment and downregulated leukocyte adhesion molecules [such as *Selplg* (selectin P ligand), *Pecam1* (platelet endothelial cell adhesion molecule), integrin subunits *Itgal* (Integrin Subunit Alpha L), *Itga4* (Integrin Subunit Alpha 4), *Itgb1* (Integrin Subunit Beta 1)], and chemokine receptors (such as *Ccr5* [C-C chemokine receptor type 5), *Cxcr2+4* (C-X-C Motif chemokine receptor types 2 + 4), and *Cx3cr1* (C-X3-C Motif Chemokine Receptor 1)], as revealed by gene expression profiling (**Figure 2E**). Similarly, blood Ly6C^high^ monocytes also underwent phenotypic changes in response to colchicine and downregulated leukocyte adhesion molecules (such as *Sell* (Selectin L, CD62L) and integrin subunit *Itgal*) and chemokine receptors (such as *Cxcr4*) (**Figure 2F**). We next tested *in vivo* how these colchicine-induced phenotypic changes impact leukocyte recruitment. We treated transgenic *Ubc-GFP* mice (all leukocytes are GFP^high^) with colchicine for seven days and then retrieved both monocytes and neutrophils from these mice (experimental setup outlined in **Figure 3A**). These GFP^high^ leukocytes were then adoptively transferred into atherosclerotic *Apoe^-/-^* recipient mice, which were not treated with colchicine. In this setting, only transferred blood monocytes and neutrophils, but not endothelial cells, were exposed to colchicine. 24h after the transfer we enumerated GFP^high^ leukocytes inside atherosclerotic plaques using flow cytometry and found that colchicine-exposed neutrophils and monocytes were recruited less in comparison to vehicle-exposed neutrophils and monocytes (**Figures 3B, C**, **Supplementary Figure 3**). These data indicate that colchicine dampened recruitment of neutrophils and monocytes into plaques primarily by altering the recruitment profile of these immune cells. Leukocyte subset numbers were unchanged in the blood and bone marrow (**Figure 4**).

### Colchicine Treatment Impacted Neither Transition of Monocytes to Macrophages nor Macrophage Proliferation

**Figure 5:**
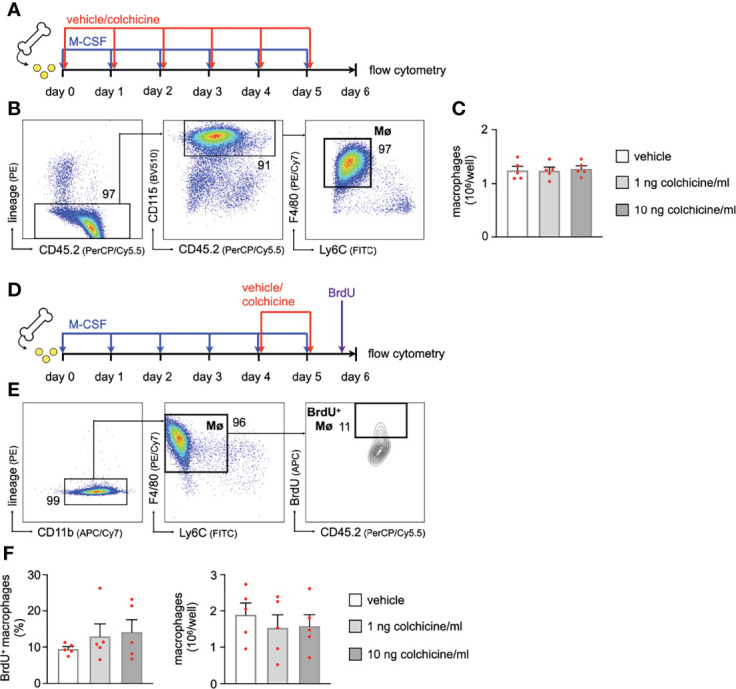
Colchicine treatment impacts neither macrophage precursor differentiation nor macrophage proliferation. **(A)** Experimental scheme for precursor differentiation into macrophages (Fig. 6B+C). In brief, bone marrow cells were retrieved from one femur and cultured with M-CSF (macrophage colony-stimulating factor) for 6 days to generate bone marrow-derived macrophages (BMDM). Either vehicle or colchicine was added once every 24h starting immediately after seeding. **(B)** Flow cytometric gating and **(C)** quantification of macrophage (Mø) numbers 6 days after either vehicle or colchicine exposure (*n*=5 per group, each *n* represents one donor animal; repeated measures one-way ANOVA with Dunnett’s multiple comparisons test). **(D)** Experimental scheme for macrophage proliferation (Fig. 6E+F). In brief, bone marrow cells were retrieved from one femur and cultured with M-CSF (macrophage colony-stimulating factor) for 6 days to generate bone marrow-derived macrophages (BMDM). Either vehicle or colchicine was added once every 24h starting 4d after seeding. BrdU (bromodeoxyuridine) was administered 2h before the harvest (day 6 after seeding). **(E)** Flow cytometric gating and **(F)** quantification of BrdU^+^ macrophage (BrdU^+^ Mø) frequencies (Friedman test followed by Dunn’s multiple comparisons test) and total macrophage numbers 48h after either vehicle or colchicine exposure (repeated measures one-way ANOVA with Dunnett’s multiple comparisons test). *n*=5 per group (each *n* represents one donor animal). Data are presented as mean+s.e.m. Numbers next to gates indicate population frequencies (%).

Apart from reducing uptake, we tested whether colchicine – a known anti-mitotic agent with anti-proliferative effects – also prevents accumulation of plaque macrophages by 1) inhibiting differentiation/maturation of recruited monocytes into macrophages or 2) reducing *in-situ* proliferation of plaque macrophages. To this end, we seeded murine bone marrow cells (retrieved from flushed femurs of wild-type mice) and incubated them with M-CSF (macrophage colony-stimulating factor) to generate bone marrow-derived macrophages (BMDM). In kinetic experiments, we found the strongest surge in macrophage numbers around days 3 to 5 (**Supplementary Figures 4A–C**). To interfere with macrophage generation, we added either vehicle or colchicine early on starting immediately after seeding (doses: 1 and 10 ng colchicine/ml; a 100 ng colchicine/ml dose was found to be cytotoxic) for six days (**Figure 5A**). We found macrophage numbers to be unchanged between vehicle and different doses of colchicine (**Figures 5B, C**). Experiments in which we added colchicine at a later time point and/or for shorter periods revealed the same results and again did not show differences in macrophage numbers (**Supplementary Figures 5A–H**). These data suggest that colchicine may not impact maturation/differentiation of monocyte progenitors/monocytes into macrophages. To assess a potential effect of colchicine on macrophage proliferation, we first generated BMDM and subsequently added colchicine on day 4 for 48h (**Figure 5D**). Using cell cycle analyses, we found that the macrophage proliferation rate (BrdU (bromodeoxyuridine) incorporation) did not differ between groups (**Figures 5E, F**). Consequently, colchicine did not change macrophage numbers over vehicle (**Figure 5F**) on day 6. Similar results regarding proliferation were obtained when we added colchicine earlier and/or used shorter incubation periods (**Supplementary Figures 6A–F**). Taken together, these data suggest that colchicine – used in dosages that resemble the colchicine plasma concentration range in treated patients (15) – may not reduce plaque macrophage accumulation by i) preventing the transition of monocytes into macrophages or by ii) impacting macrophage proliferation.

### Colchicine Treatment Curtailed Leukocyte Accumulation in Atherosclerotic Aortas Also in Post-MI Accelerated Atherosclerosis

**Figure 6:**
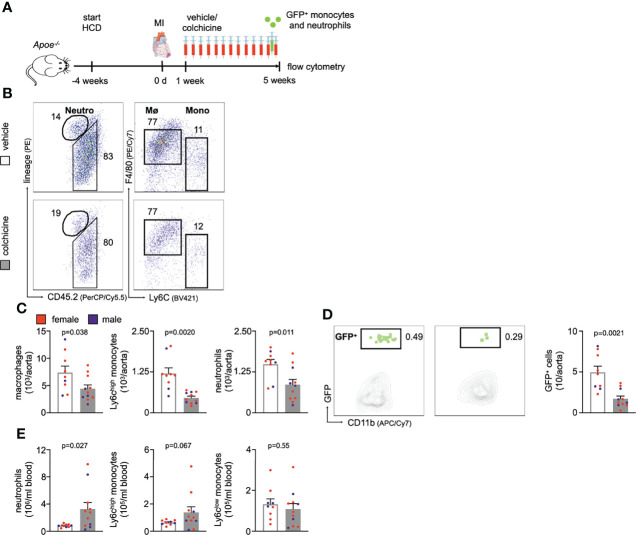
Colchicine treatment reduces vascular inflammation in post-myocardial infarction accelerated atherosclerosis. **(A)** Experimental scheme for **Figures 6B-E**. In brief, *Apoe^-/-^* mice on a high cholesterol diet (HCD) were subjected to myocardial infarction (MI) and treated with either vehicle or colchicine for four weeks starting one week after induction of MI. **(B)** Flow cytometric gating and **(C)** quantification of leukocyte subsets in atherosclerotic aortas in vehicle- vs. colchicine-treated *Apoe^-/-^* mice which were infarcted five weeks prior (*n*=9-11 per group, 64-67% female, Student’s *t*-test for macrophages and neutrophils, Welch’s *t*-test for monocytes). Neutro: neutrophils, Mø: macrophages and Mono: monocytes **(D)** Flow cytometric gating and quantification of GFP^high^ myeloid cells (GFP^+^) in atherosclerotic aortas 24h after adoptive transfer of GFP^high^ monocytes and neutrophils into vehicle- vs. colchicine-treated *Apoe^-/-^* mice which were infarcted five weeks prior (*n*=9-11 per group, 64-67% female, Welch’s *t*-test). **(E)** Quantification of blood leukocyte subsets in vehicle- vs. colchicine-treated *Apoe^-/-^* mice which were infarcted five weeks prior (*n*=9-11 per group, 64-67% female, Welch’s *t*-test for neutrophils, Mann-Whitney *U*-test for Ly6C^high^ monocytes, Student’s *t*-test for Ly6C^low^ monocytes). Data are presented as mean+s.e.m. Numbers next to gates indicate population frequencies (%). Dots within bar plots show the gender of the mice with a color code: purple (male) and red (female).

The above performed experiments explored how colchicine treatment impacts early atherosclerosis (atherogenesis), i.e. primary prevention. We next probed whether these beneficial effects also occur in secondary prevention, i.e. in the setting of prior MI as investigated in the COLCOT trial. We surgically induced MI (permanent left anterior descending coronary artery ligation) in atherosclerotic mice (**Figure 6A**) and administered either vehicle or colchicine for 4 weeks starting one week after MI. We then excised atherosclerotic aortas, enumerated leukocytes, and found that colchicine treatment reduced numbers of macrophages, Ly6C^high^ monocytes, and neutrophils (**Figures 6B, C**, **Supplementary Figure 7**). Using adoptive transfer, we tested whether colchicine also reduced uptake of monocytes and neutrophils and found fewer GFP^high^ myeloid cells inside atherosclerotic aortas in the treatment group in post-MI accelerated atherosclerosis (**Figure 6D**). Exploring the blood compartment, we found higher numbers of blood neutrophils (**Figure 6E**). These data suggest that colchicine treatment reduced inflammatory leukocyte recruitment in post-MI accelerated (**Figure 6**) as well as primary atherosclerosis (**Figures 1** **–** **4**).

### Colchicine Treatment Limited Expansion of Myeloid Cells Also in Acute and Chronic Cardiac Inflammation

Finally, we tested colchicine also in the setting of cardiac inflammation (16). To this end, wild-type mice were subjected to experimental MI (permanent left anterior descending coronary artery ligation) and treated with either high dose colchicine or vehicle starting two hours after induction of cardiac ischemia (**Supplementary Figure 8A**). Three days after MI, we enumerated leukocyte numbers inside the ischemic cardiac area using flow cytometry (**Supplementary Figure 8B**). We found fewer neutrophils in colchicine treated mice with MI while numbers for other myeloid cells remained unchanged (**Supplementary Figure 8C**). Next, we extended our investigations from acute MI to chronic MI/chronic ischemic cardiomyopathy with chronic heart failure. Here, we infarcted *Apoe^-/-^* mice and started administering vehicle or colchicine 7 days after MI for 4 weeks (**Figure 6A**). Adoptive transfer experiments revealed reduced uptake of adoptively transferred GFP^+^ cells into infarcted hearts in the colchicine group (**Supplementary Figure 8D**). Consequently, numbers of cardiac inflammatory monocytes and macrophages decreased in colchicine treated mice (**Supplementary Figure 8E**). To conclude, colchicine reduced numbers of cardiac inflammatory leukocytes within infarcted hearts, especially affecting neutrophils in the acute setting, and monocytes/macrophages in the chronic phase after MI.

## Discussion

Colchicine, derived from the plant extract of *Colchicum autumnale*, is a broadly available, inexpensive, orally administered drug with multiple mechanisms of action, some of which are still under investigation (9, 15, 17, 18). The primary intracellular targets of colchicine are microtubules which are essential components of the cellular cytoskeleton. Microtubules play a pivotal role in maintaining cell shape, intracellular trafficking, cytokine secretion, cell migration, and cell division (9). At low doses, colchicine prevents growth of microtubules, while it supports microtubule depolymerization at high doses. Colchicine’s effect on tubulin disruption prevents the formation of the inflammasome which results in less secretion of pro-inflammatory cytokines and impaired neutrophil function (9, 19). Given this background, colchicine was explored as a novel anti-inflammatory treatment strategy in patients with coronary artery disease (CAD). In the LoDoCo trial, low-dose colchicine treatment reduced cardiovascular events in patients with stable CAD (20). These results were confirmed in the LoDoCo2 trial which reported a 31% relative risk reduction of cardiovascular events in stable CAD patients treated with colchicine compared to placebo (8). Regarding acute coronary syndromes, the COLCOT trial reported that among patients with recent MI, colchicine at a dose of 0.5 mg daily reduced cardiovascular outcomes over placebo, an effect that was mainly driven by a lower incidence of strokes and urgent hospitalizations for angina leading to coronary revascularization (7).

Although these results are encouraging, we do not fully understand the underlying mechanisms for the cardiovascular benefits of colchicine. In this study we provide evidence that colchicine reduces vascular inflammation and dampens progression of atherosclerosis. Causally, we found that colchicine decreased accumulation of inflammatory leukocytes inside atherosclerotic plaques which in turn altered the overall plaque character. In line with our study, a recent report showed that colchicine decreased plaque vulnerability with reductions in plaque inflammation, medial fibrosis and vascular remodeling in atherosclerotic rabbits (21). According to our data, colchicine reduced expansion of inflammatory leukocytes by preventing influx of inflammatory monocytes and neutrophils from the blood into aortic plaques. Previously, it was described that colchicine can act on both endothelial cells and neutrophils: in endothelial cells colchicine reduced expression of E-selectin, in neutrophils colchicine decreased expression of L-selectin and neutrophil extracellular trap formation (22, 23). In line, we also found that blood monocytes/neutrophils and plaque endothelial cells underwent phenotypic changes in response to colchicine. However, colchicine seems to affect blood leukocytes stronger than endothelial cells. While transcriptomic profiling of FACS-purified plaque endothelial cells revealed only mild colchicine-induced alterations, we found substantial changes in blood neutrophils and Ly6C^high^ monocytes. We determined the consequences of reduced myeloid cell accumulation in colchicine treated mice using qPCR. Whether the observed reduction in expression levels led to reduced protein levels remains to be investigated. In sum, these data demonstrate that colchicine mitigates blood leukocyte recruitment into atherosclerotic aortas predominantly by silencing blood neutrophil and monocyte activation, suggesting a largely endothelial cell-independent effect of colchicine on inflammatory leukocyte recruitment. However, we cannot exclude that colchicine also impacts the recruitment profile of endothelial cells beyond adhesion molecule and chemokine expression (24).

Further research is needed to decipher whether colchicine exerts its action 1) exclusively on circulating blood monocytes and neutrophils or 2) on monocytes and neutrophils in other compartments prior to egress into the blood. The fact that we did not observe a retention of neutrophils and monocytes in the bone marrow (their numbers were unchanged), however, argues for an effect that is mediated in the circulation. Moreover, further investigations that explore how exactly colchicine leads to downregulation of adhesion molecules and chemokine receptors on monocytes and neutrophils are needed. One may speculate that colchicine-induced microtubules’/cytoskeleton alterations affect cell adhesion molecules since both are known to bidirectionally interact (25).

Apart from increased monocyte recruitment, macrophages may also expand inside plaques through accelerated conversion/differentiation of recruited monocytes and/or *in-situ* proliferation (26). We cultured mouse whole bone marrow cells with M-CSF – a cytokine that causes bone marrow cells to differentiate into macrophages – and added either vehicle or colchicine early on. Our kinetics experiments revealed that macrophage numbers were not at any time point affected by colchicine treatment. These findings are in line with a prior report where colchicine also failed to influence monocyte differentiation (27). We next tested the possibility that colchicine dampened accumulation of plaque macrophages by reducing *in-situ* macrophage proliferation. Although described for vascular smooth muscle cells (28), colchicine did not influence macrophage proliferation. Taken together, colchicine does not seem to impact i) monocyte trans-differentiation into macrophages or ii) macrophage proliferation in *in-vitro* experiments using colchicine concentrations comparable to clinical settings.

Colchicine was also tested to treat acute coronary syndromes in humans. In this context, high-dose colchicine was administered over a short period starting early after onset of chest pain/MI. While one study revealed smaller infarct sizes in MI patients treated with colchicine, two other recent trials failed to document a clear benefit of colchicine in the acute stetting (29–31). This motivated us to investigate colchicine also in acute and chronic MI. Our experiments reveal that colchicine – as in the setting of atherosclerosis – reduced accumulation of cardiac inflammatory myeloid cells, indicating the operation of convergent mechanisms (reduced uptake of inflammatory leukocytes).

## Conclusion

Taken together, our data suggest that colchicine – an inhibitor of microtubule assembly with anti-inflammatory properties – reduces vascular inflammation and plaque progression by silencing monocyte and neutrophil activation and thereby preventing uptake of these cells into atherosclerotic plaques. Our data shed new light on the mechanism by which colchicine reduces the risk of cardiovascular complications.

## Data Availability Statement

The original contributions presented in the study are included in the article/**Supplementary Material**. Further inquiries can be directed to the corresponding author.

## Ethics Statement

The animal study was reviewed and approved by the local animal care committee (Regierung von Oberbayern).

## Author Contributions

HSa and TK generated the hypothesis and conceived the project. HSa, TK, UM-L, CM, A-CS, and AM designed experiments. HSa, UM-L, CM, A-CS, AM, JHi, JHe, DK, CG, and AD performed experiments. HSa, UM-L, CM, A-CS, and AM analyzed and interpreted data. XL performed MI surgeries. TK and HSc provided intellectual input and edited the manuscript. HSa, UM-L, CM, A-CS, and AM made the figures. HSa, CM, and UM-L wrote the manuscript, which was approved by all authors.

## Funding

HSa has received funding from the European Research Council under the European Union’s Horizon 2020 Research and Innovation Programme (STRATO, grant agreement No. 759272), the “Else-Kröner-Fresenius-Stiftung” (2020_EKSE.07) and the “Deutsche Herzstiftung” (F/28/17). This study was further supported by the “Deutsche Forschungsgemeinschaft (DFG)” (SA 1668/5-1 and 470462396). TK is supported by the Corona Foundation [Junior Research Group *Translational Cardiovascular Genomics*) and the “Deutsche Forschungsgemeinschaft (DFG)” (CRC 1123 (B02)]. Additionally, we gratefully acknowledge the support of the German Federal Ministry of Education and Research (BMBF), to HSc, within the framework of ERA-NET on Cardiovascular Disease (Druggable-MI-genes: 01KL1802) and the scheme of target validation (BlockCAD: 16GW0198K), as well as the German Centre of Cardiovascular Research (DZHK) Munich Heart Alliance, within the framework of the e:Med research and funding concept (AbCD-Net: 01ZX1706C). The British Heart Foundation (BHF)/German Centre of Cardiovascular Research (DZHK) Collaboration, for which we are co-applicants, has also provided support and funding. HSc has received further support from the German Research Foundation (DFG) as part of the “Sonderforschungsbereich” 1123 (B02) and the “Sonderforschungsbereich” TRR 267 (B05). Bavarian State Ministry of Health and Care also funded this work as part of “DigiMed Bayern” (grant No: DMB-1805-0001).

## Conflict of Interest

HSa reports grants from the European Research Council, the “Else-Kröner-Fresenius-Stiftung”, the “Deutsche Herzstiftung” and the “Deutsche Forschungsgemeinschaft” during the conduct of the study. HSa received lecture fees from Novo Nordisk. HSc reports personal fees from MSD Sharp & Dohme, personal fees from AMGEN, personal fees from Bayer Vital GmbH, personal fees from Boehringer Ingelheim, personal fees from Daiichi-Sankyo, personal fees from Novartis, personal fees from Servier, personal fees from Brahms, personal fees from Bristol-Myers Squibb, personal fees from Medtronic, personal fees from Sanofi Aventis, personal fees from Synlab, personal fees from Pfizer, grants and personal fees from Astra-Zeneca and personal fees from Vifor, outside the submitted work. HSc and TK are named inventors on a patent application for prevention of restenosis after angioplasty and stent implantation outside the submitted work. TK received lecture fees from Bayer AG, Pharmaceuticals.

The remaining authors declare that the research was conducted in the absence of any commercial or financial relationships that could be construed as a potential conflict of interest.

## Publisher’s Note

All claims expressed in this article are solely those of the authors and do not necessarily represent those of their affiliated organizations, or those of the publisher, the editors and the reviewers. Any product that may be evaluated in this article, or claim that may be made by its manufacturer, is not guaranteed or endorsed by the publisher.
