# Supplementary material for: Colchicine Impacts Leukocyte Trafficking in Atherosclerosis and Reduces Vascular Inflammation
Source: Front Immunol. 2022 Jul 4;13:898690. doi: 10.3389/fimmu.2022.898690 (PMC9289246; doi:10.3389/fimmu.2022.898690)
Supplement: Supplementary file 1 [file DataSheet_1.docx]

Supplementary Material

# Supplementary Figures


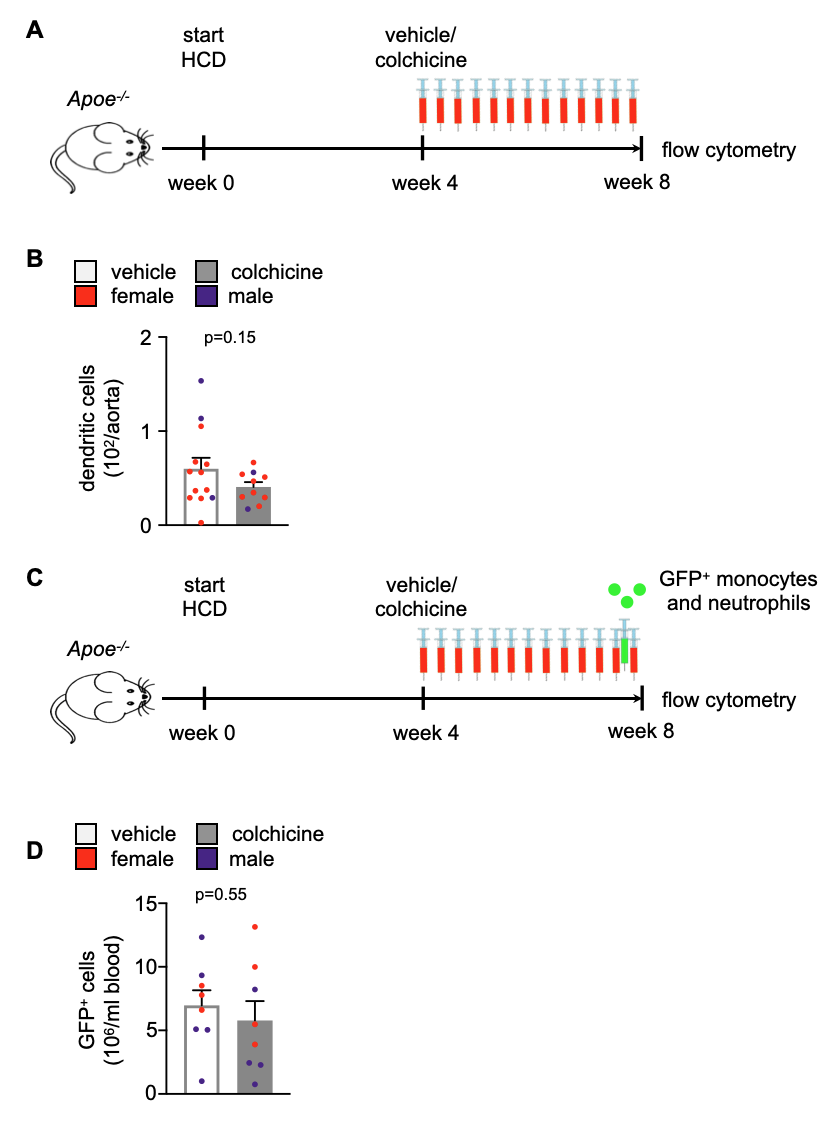

 **Supplementary Figure 1.** **(A)** Experimental scheme for **Figure 1**, **Figure 2 (B-F)** and **Figure 4**. *Apoe^-/-^* mice were subjected to a cholesterol-enriched diet (high cholesterol diet, HCD) for a period of 8 weeks and treated daily with intraperitoneal injections of either vehicle or colchicine (0.25 mg/kg BW) in the last 4 weeks of HCD. **(B)** Flow cytometric quantification of dendritic cells in atherosclerotic aortas in vehicle- vs. colchicine- treated Apoe^-/-^ mice (n=13-10 per group, 70 - 80% female, Welch’s t-test). **(C)** Experimental scheme for **Figure 2A**. In addition to the treatment described in **(A)**, *Apoe^-/-^* mice were intravenously injected with GFP^+^ monocytes and neutrophils 24 hours before the harvest. **(D)** Quantification of GFP^high^ myeloid cells in blood, 24h after adoptive transfer of GFP^high^ monocytes and neutrophils into vehicle- vs. colchicine-treated *Apoe^-/-^* mice (*n*=8 per group, 38-50% female, Student’s *t-*test). Dots within bars show the gender of the mice with a color code: purple (male) and red (female).

**
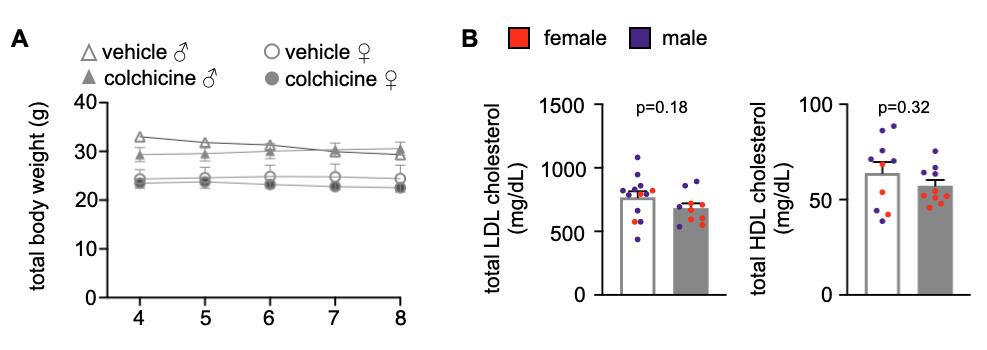


Supplementary Figure 2.** Quantification of **(A)** weight development within the treatment period (x-axis indicates time course between weeks 4 and 8 of treatment; *n*=4-6 per group, two-way ANOVA with Dunnett’s multiple comparisons test – adjusted *P*-value >0.15 for all comparisons), **(B)** plasma LDL (low-density lipoprotein) cholesterol and HDL (high-density lipoprotein) cholesterol levels in vehicle- vs. colchicine-treated *Apoe^-/-^* mice (*n*=10 per group, 40-60% female, Student’s *t*-test). Data are presented as mean+s.e.m. Dots within bars show the gender of the mice with a color code: purple (male) and red (female).


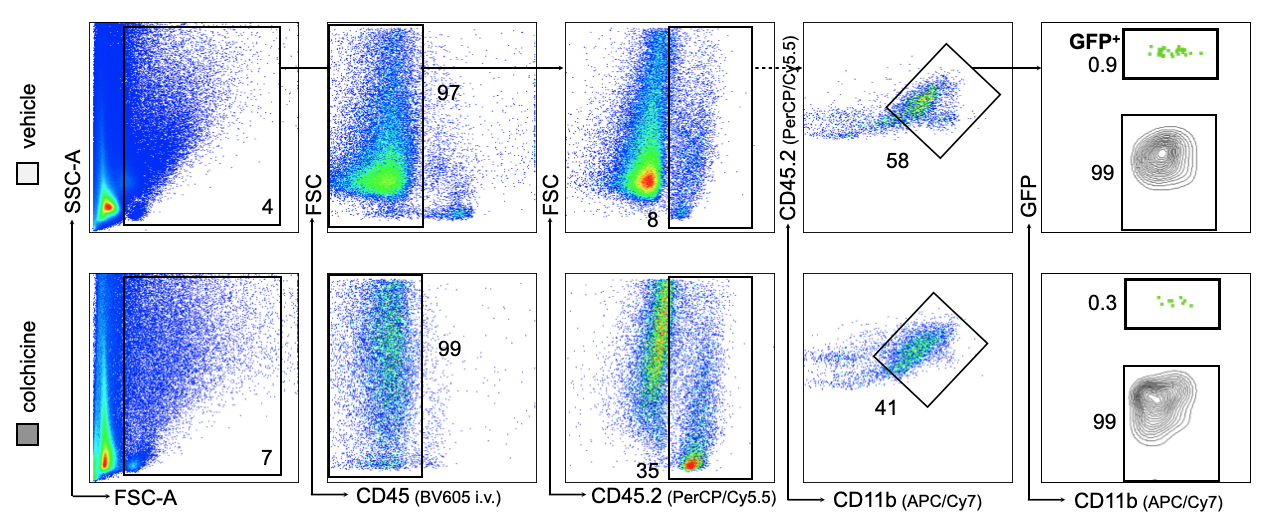


**Supplementary Figure 3.** Flow cytometric gating of GFP^high^ myeloid cells (GFP^+^) in atherosclerotic aortas 24h after adoptive transfer of either vehicle- or colchicine-exposed GFP^high^ monocytes and neutrophils into *Apoe^-/-^* mice, referring to Figure 3 (*n*=10 per group, 60% female, Mann-Whitney *U*-test). Data are presented as mean+s.e.m. Numbers next to gates indicate population frequencies (%).


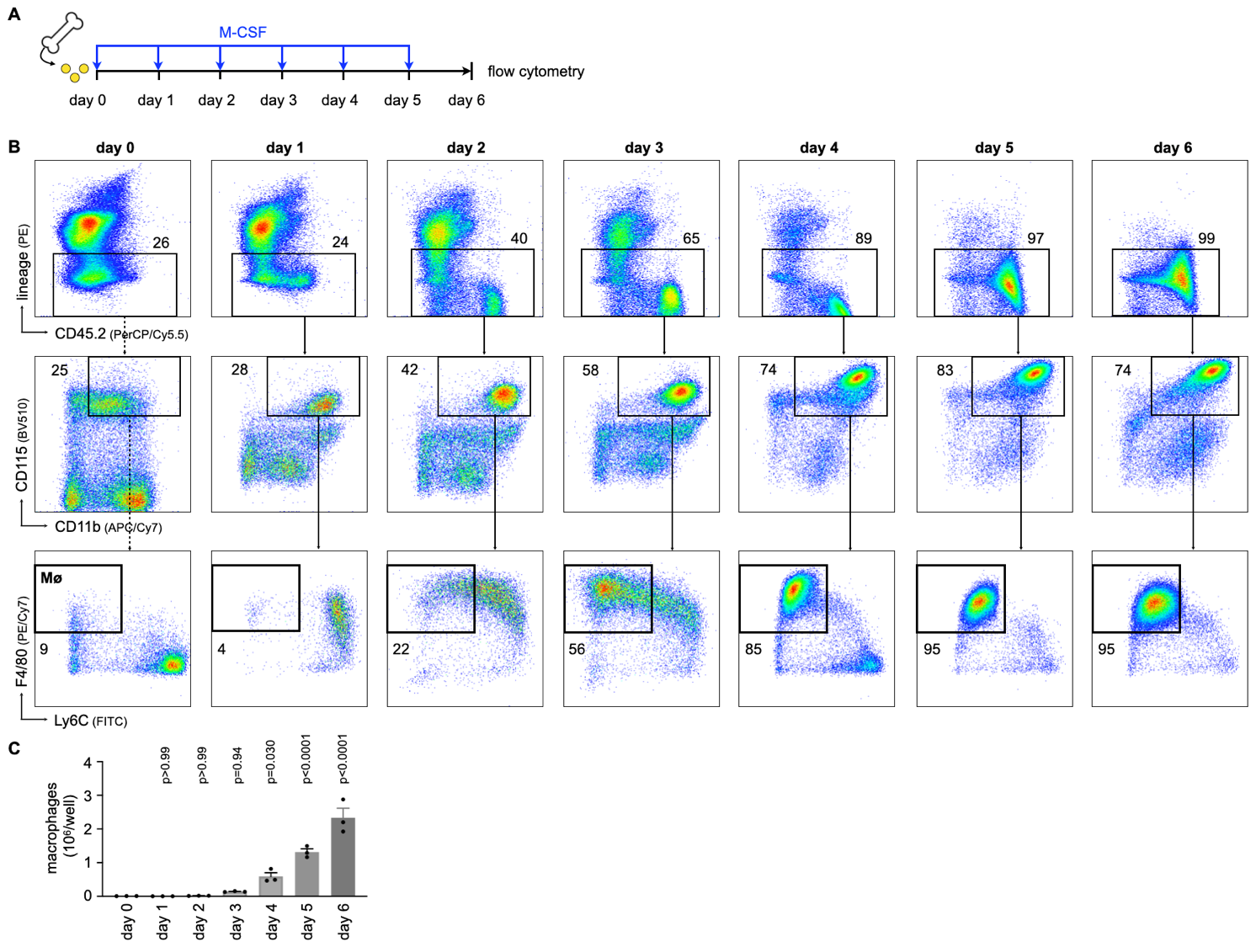


**Supplementary Figure 4. Macrophage numbers rise from day 3 onward in cultured whole bone marrow cells incubated with M-CSF. (A)** Experimental scheme for culturing of mouse whole bone marrow cells in the presence of M-CSF (macrophage colony-stimulating factor) to generate bone marrow-derived macrophages (Mø). **(B)** Flow cytometric gating and **(C)** quantification of macrophages in kinetic experiments (*n*=3 per group, each *n* represents one donor animal, repeated measures one-way ANOVA with Dunnett’s multiple comparisons test). Data are presented as mean+s.e.m. Numbers next to gates indicate population frequencies (%).

**
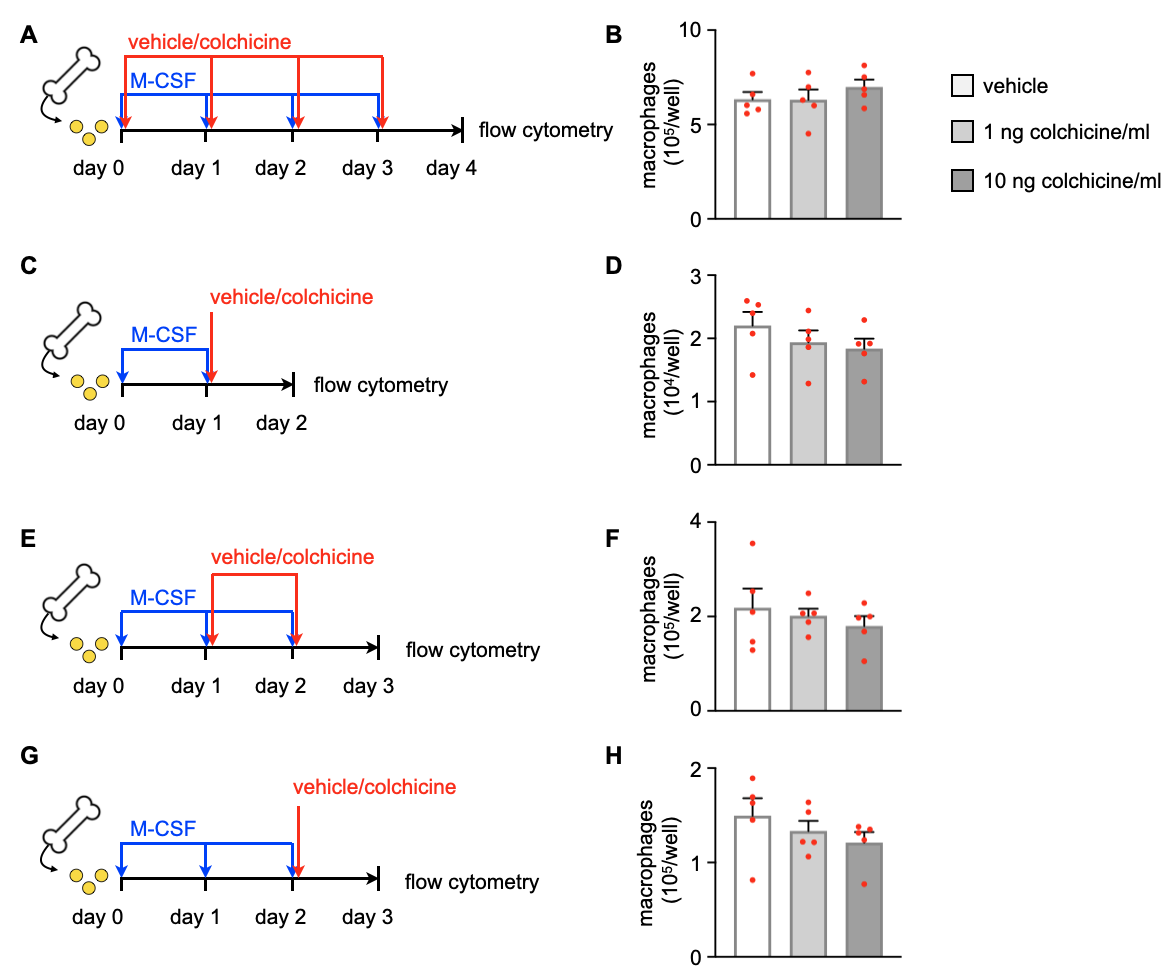
**

**Supplementary Figure 5. Colchicine treatment does not impact macrophage precursor differentiation. (A,C,E,G)** Experimental schemes for precursor differentiation into macrophages. In brief, bone marrow cells were retrieved from one femur and cultured with M-CSF (macrophage colony-stimulating factor) for respective days to generate bone marrow-derived macrophages (BMDM). Either vehicle or colchicine was added once every 24 hours as indicated. **(B,D,F,H)** Quantification of macrophage numbers after either vehicle or colchicine exposure (*n*=5 per group, each *n* represents one donor animal; repeated measures one-way ANOVA with Dunnett’s multiple comparisons test or Friedman test followed by Dunn’s multiple comparisons test as appropriate). Data are presented as mean+s.e.m.


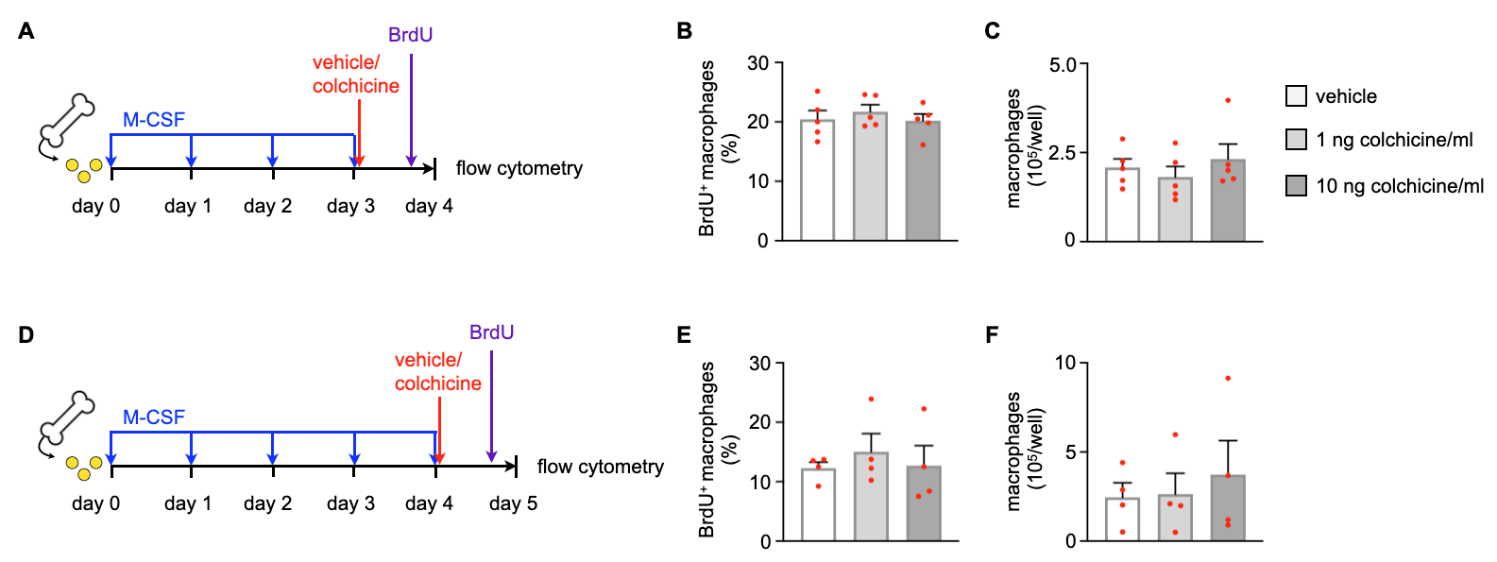


**Supplementary Figure 6. Colchicine treatment does not impact macrophage proliferation. (A,D)** Experimental schemes for macrophage proliferation. In brief, bone marrow cells were retrieved from one femur and cultured with M-CSF (macrophage colony-stimulating factor) for respective days to generate bone marrow-derived macrophages (BMDM). Either vehicle or colchicine was added once every 24h starting as indicated. BrdU (bromodeoxyuridine) was administered 2h before the harvest. **(B,C,E,F)** Quantification of BrdU^+^ macrophage frequencies and total macrophage numbers after either vehicle or colchicine exposure (*n*=4-5 per group, each *n* represents one donor animal; repeated measures one-way ANOVA with Dunnett’s multiple comparisons test or Friedman test followed by Dunn’s multiple comparisons test as appropriate). Data are presented as mean+s.e.m.


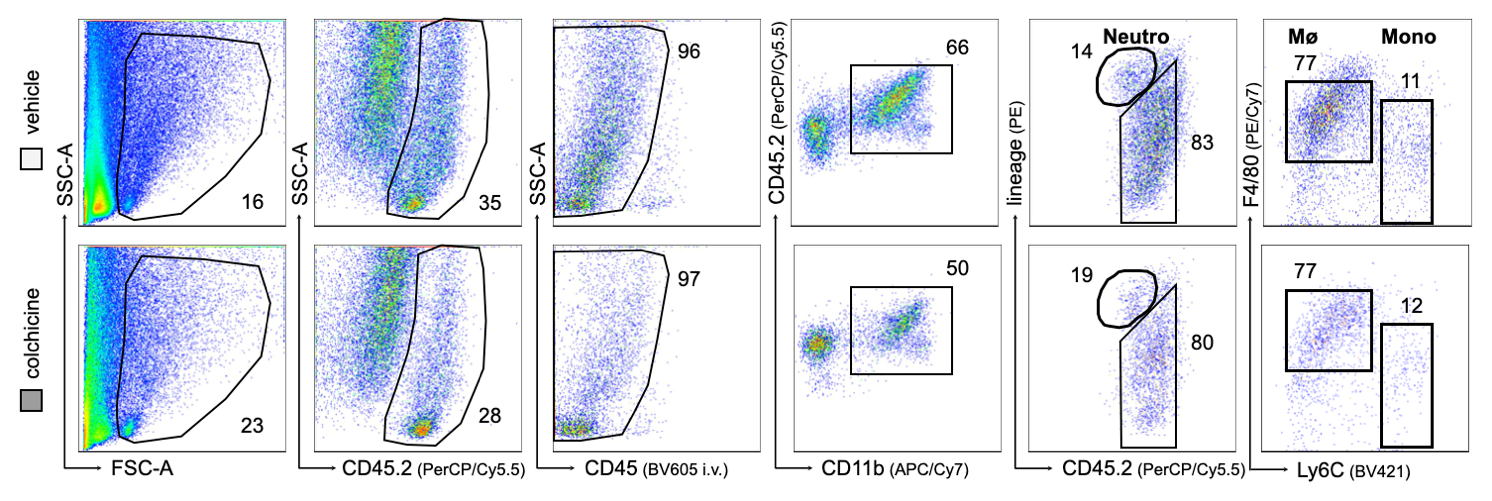


**Supplementary Figure 7.** Complete flow cytometric gating of leukocyte subsets in atherosclerotic aortas in vehicle- vs. colchicine-treated *Apoe^-/-^* mice which were infarcted five weeks prior, referring to Figure 6 (*n*=9-11 per group, 64-67% female, Student’s *t*-test for macrophages and neutrophils, Welch’s *t*-test for monocytes). Neutro: neutrophils, Mø: macrophages, Mono: monocytes.


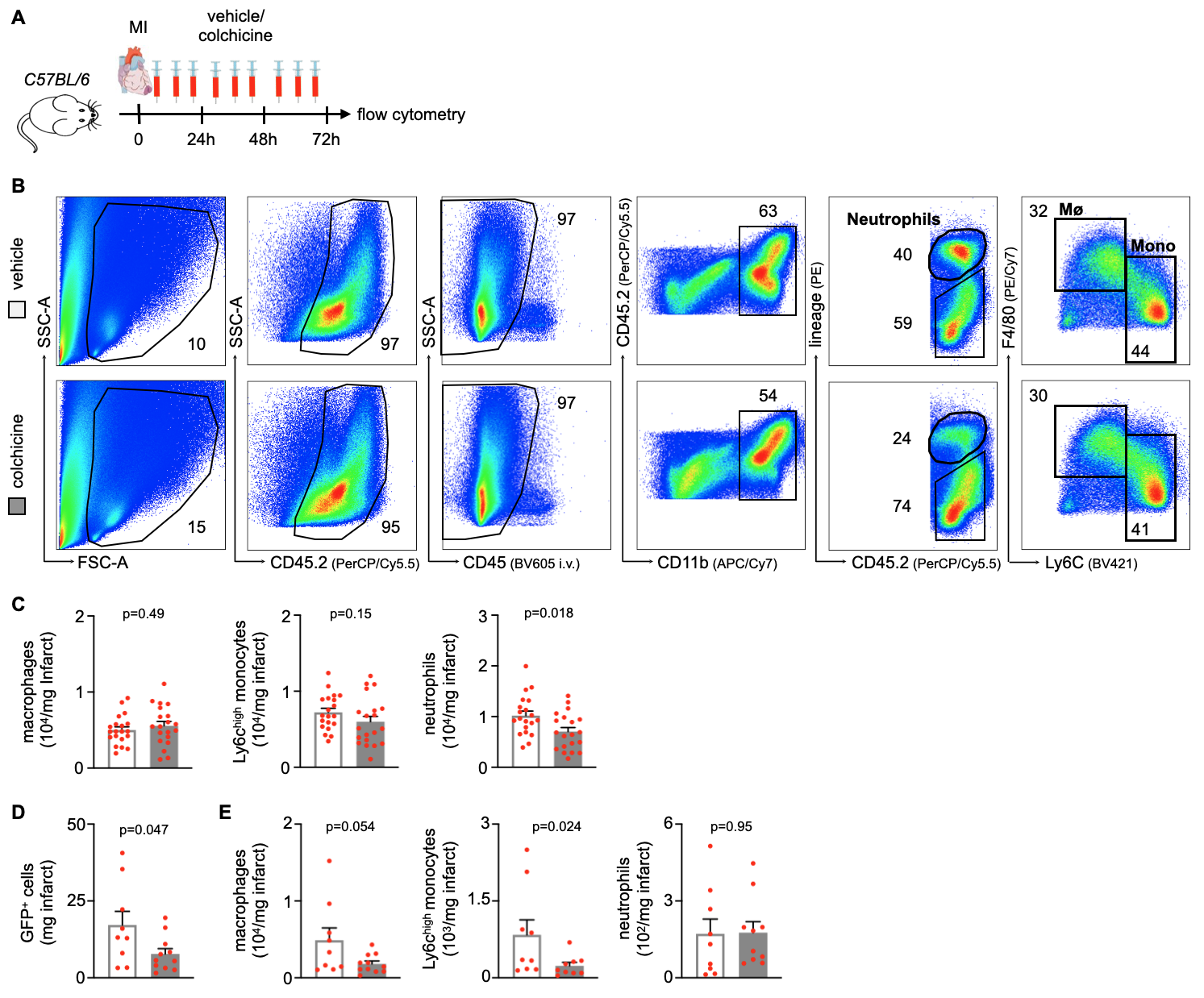


**Supplementary Figure 8: Colchicine treatment limits expansion of myeloid cells in acute and chronic cardiac inflammation. (A)** Experimental scheme. In brief, C57BL/6 J mice were subjected to myocardial infarction (MI) and treated with either vehicle or colchicine daily for three days, starting two hours after induction of MI. **(B)** Flow cytometric gating and **(C)** quantification of leukocyte subsets in infarcted heart tissue in vehicle- vs. colchicine-treated mice (*n*=20 per group, 100% female, Student’s *t*-test). **(D,E)** *Apoe^-/-^* mice on a high cholesterol diet (HCD) were subjected to myocardial infarction (MI) and treated with either vehicle or colchicine for four weeks starting one week after induction of MI. 24h prior to harvest, GFP^+^ monocytes and neutrophils were adoptively transferred into the vehicle- vs. colchicine-treated *Apoe^-/-^* mice. Quantification of **(D)** GFP^+^ cells and **(E)** leukocyte subsets in infarcted heart tissue 24h after adoptive transfer (*n*=9-11 per group, 64-67% female, Student’s *t*-test for GFP^+^ cells, macrophages and neutrophils, Mann-Whitney *U*-test for Ly6C^high^ monocytes). Data are presented as mean+s.e.m. Numbers next to gates indicate population frequencies (%). Mø: macrophages, Mono: monocytes.

# Supplementary Tables

**Supplementary Table S1.** TaqMan probes used for real-time qPCR.

| **Gene of interest** | **TaqMan probe** |
| --- | --- |
| *Ccl2* | Mm00441242_m1 |
| *Ccr1* | Mm00438260_s1 |
| *Ccr2* | Mm99999051_gH |
| *Ccr5* | Mm01963251_s1 |
| *Col1a2* | Mm00483888_m1 |
| *Col3a1* | Mm01254476_m1 |
| *Csf1* | Mm00432686_m1 |
| *Cx3cl1* | Mm00436454_m1 |
| *Cxcl1* | Mm04207460_m1 |
| *Cxcl2* | Mm00436450_m1 |
| *Cxcl12* | Mm00445553_m1 |
| *Cx3cr1* | Mm02620111_s1 |
| *Cxcr2* | Mm99999117_s1 |
| *Cxcr4* | Mm01292123_m1 |
| *Gapdh* | Mm99999915_g1 |
| *Icam1* | Mm00516023_m1 |
| *Icam2* | Mm00494862_m1 |
| *Il1b* | Mm00434228_m1 |
| *Il6* | Mm00446190_m1 |
| *Itga4* | Mm01277951_m1 |
| *Itgal* | Mm00801807_m1 |
| *Itgam* | Mm00434455_m1 |
| *Itgb1* | Mm01253230_m1 |
| *Itgb2* | Mm00434513_m1 |
| *Mmp3* | Mm00440295_m1 |
| *Mmp9* | Mm00442991_m1 |
| *Mmp10* | Mm01168399_m1 |
| *Pecam1* | Mm01242576_m1 |
| *Sele* | Mm00441278_m1 |
| *Sell* | Mm00441291_m1 |
| *Selp* | Mm01295931_m1 |
| *Selplg* | Mm01204601_m1 |
| *Tgfb1* | Mm01178820_m1 |
| *Tnf* | Mm00443258_m1 |
| *Vcam1* | Mm01320970_m1 |
